# Construction and Verification of Risk Predicting Models to Evaluate the Possibility of Venous Thromboembolism After Robot-Assisted Radical Prostatectomy

**DOI:** 10.1245/s10434-022-11574-5

**Published:** 2022-03-22

**Authors:** Xu Cheng, Lizhi Zhou, Wentao Liu, Yijian Li, Mou Peng, Yinhuai Wang

**Affiliations:** Department of Urology, The Second Xiangya Hospital, Central South University, Changsha, 410011 Hunan China

## Abstract

**Background:**

Venous thromboembolism (VTE) is the second leading cause for death of radical prostatectomy. We aimed to establish new nomogram to predict the VTE risk after robot-assisted radical prostatectomy (RARP).

**Methods:**

Patients receiving RARP in our center from November 2015 to June 2021, were enrolled in study. They were randomly divided into training and testing cohorts by 8:2. Univariate and multivariate logistic regression (model A) and stepwise logistic regression (model B) were used to fit two models. The net reclassification improvement (NRI), integrated discrimination improvement (IDI), and receiver operating characteristic (ROC) curve were used to compare predictive abilities of two new models with widely used Caprini risk assessment (CRA) model. Then, two nomograms were constructed and received internal validation.

**Results:**

Totally, 351 patients were included. The area under ROC of model A and model B were 0.967 (95% confidence interval: 0.945–0.990) and 0.978 (95% confidence interval: 0.960–0.996), which also were assayed in the testing cohorts. Both the prediction and classification abilities of the two new models were superior to CRA model (NRI > 0, IDI > 0, *p* < 0.05). The C-index of Model A and Model B were 0.968 and 0.978, respectively. For clinical usefulness, the two new models offered a net benefit with threshold probability between 0.08 and 1 in decision curve analysis, suggesting the two new models predict VTE events more accurately.

**Conclusions:**

Both two new models have good prediction accuracy and are superior to CRA model. Model A has an advantage of less variable. This easy-to-use model enables rapid clinical decision-making and early intervention in high-risk groups, which ultimately benefit patients.

**Supplementary Information:**

The online version contains supplementary material available at 10.1245/s10434-022-11574-5.

Prostate cancer ranks second in cancer incidence worldwide and sixth in cancer-related mortality.^1^ Radical prostatectomy is the first-line treatment for localized and selected locally advanced prostate cancer.^2^ However, patients receiving radical prostatectomy suffer a lot from its complications, which can be a threat to their lives and brings a huge economic burden on their families.^3^

Venous thromboembolism (VTE) is composed of deep vein thromboses (DVT) and pulmonary embolism (PE), considered as the serious and sometimes fatal complication of surgery.^4^ The current study showed that VTE is the second-leading cause for the death of radical prostatectomy, resulting in a great threat to the lives of patients.^5,6^ Numerous risk factors for VTE are well documented, including prior VTE, smoking, larger prostate volume, longer operative time, higher body mass index (BMI), blood transfusion, lymph node dissection, T stage, Gleason score, and age, which may potentially promote thrombosis.^7–11^

The existing predicting tool associated with VTE (e.g., the widely used Caprini score system), despite having a degree of value in identifying the risk of VTE, however, is still not specifically used to evaluate the risk of postoperative thrombosis events in patients undergoing urological surgery. Actually, the risk of postoperative thrombosis events varies with different tumors.^12^ There is a lack of tools to predict the risk of postoperative thrombosis events in patients receiving radical prostatectomy yet.

Nomogram is a visible way to show the regression results, which can express the significance of different variables on the outcome by the length of the line. By calculating the total score, the probability of the outcome in the patient can be visually displayed.^13–15^ However, there are still limited studies on using nomograms to predict the risk of thrombosis events.^16^ In this study, we established two nomograms to predict the risk of VTE after robot-assisted radical prostatectomy (RARP). Referring to these models, we believed that doctors could identify patients with a high risk of VTE and take timely preventive measures and treatments at an early stage, which enable to help reduce the risk of postoperative thrombotic events after RARP.

## Methods

### Study Design and Participants

A total of 351 patients receiving RARP in the Xiangya Second Affiliated Hospital, Central South University from November 1, 2015, to June 1, 2021, were enrolled in this study. All of them are Han Chinese, and no separate emphasis in the Table 1. The inclusion criteria of this study were as follows: (1) adult patients who were pathologically diagnosed with prostate cancer and underwent successful RARP in our center; (2) patients with complete clinical records for the present study. The exclusion criteria were as follows: (1) patients diagnosed with occurrent VTE before RARP; (2) patients with primary or secondary coagulation disorders; (3) patients who underwent reoperation for bleeding, anastomosis fistula, etc. at the hospital admission; (4) Patients under treatment of curative anticoagulation by warfarin, heparin, etc. at hospital admission; (5) Patients with missing value of the medical records.

**Table 1:** Clinical characteristics of patients receiving radical prostatectomy

	Non-VTE (*n* = 311)	VTE (*n* = 40)	*p* value
Age (yr)	64.76 (5.63)	69.53 (4.50)	< 0.001
BMI (kg/m^2^)	23.58 (1.91)	23.58 (2.02)	0.992
Smoking	90 (28.9)	20 (50.0)	0.012
Neoadjuvant ADT	132 (42.4)	17 (42.5)	1.000
Previous history of VTE	4 (1.3)	14 (35.0)	< 0.001
^a^PSA (ng/mL)	37.63 (43.98)	46.97 (58.27)	0.226
Gleason score ≥8	96 (30.9)	29 (72.5)	< 0.001
Prostate volume (mL)	38.59 (19.30)	68.06 (24.57)	< 0.001
^b^T3/T4 stage	64 (20.6)	9 (22.5)	0.940
Preoperative			
APTT (s)	32.09 (6.83)	32.07 (6.45)	0.982
TT (s)	16.59 (1.06)	16.70 (1.59)	0.577
FIB (g/L)	2.97 (0.83)	2.92 (0.83)	0.704
Antiplatelet	9 (2.9)	4 (10.0)	0.073
^c^Operation time (min)	182.55 (59.56)	243.30 (57.85)	< 0.001
Blood transfusion	49 (15.8)	6 (15.0)	1.000
Lymph node dissection			< 0.001
Standard	24 (7.7)	4 (10.0)	
Extended	54 (17.4)	24 (60.0)	
Postoperative			
d-Dimer (mg/L)	3.23 (2.26)	4.65 (3.67)	0.001
PLT (n/L)	170.94 (47.50)	179.40 (39.13)	0.281
^d^Coagulation index	2.32 (1.96)	5.30 (1.08)	< 0.001

Data are presented as n (%) or mean (SD)

*BMI* body mass index, *ADT* androgen deprivation therapy, *VTE* venous thromboembolism, *PSA* prostate specific antigen, *APTT* activated partial thromboplastin time, *TT* thrombin time, *FIB* fibrinogen, *PLT* platelet count

^a^Preoperative total serum PSA

^b^Tumor in TNM classification

^c^Operation time included the time interval of the establishment of the surgical approach, specimen removal, and incision suture

^d^Comprehensive index in thromboelastogram reflecting coagulation status, Coagulation index (CI) = − 0.3258R − 0.1886K + 0.1224MA + 0.0759α − 7.7922

In this study, all patients were diagnosed with prostate cancer according to the histopathologic examination and the diagnosis of VTE was confirmed by a positive finding on color duplex ultrasound for DVT or computerized tomography pulmonary angiography (CTPA) for PE. All ultrasound/CTPA images were explained by two senior radiologists. We undertook a radical screening strategy to identify VTE. D‐Dimer test was given to all patients after RARP, lower limb ultrasound was given when patients with positive D‐Dimer test, and ultrasound/CTPA was given in patients with VTE-related symptoms. These examinations were performed with the permission of patients.

All patients were randomly distributed to the training and testing cohort with 7:3 by using computer-generated random numbers.^17^ Finally, patients were allocated into the training cohort (*n* = 246) and the testing cohort (*n* = 105), respectively. In addition, VTE event was an endpoint of the one-month follow-up after surgery, and the above patients were finally divided into the VTE and non-VTE groups.

### Data Collection

Candidate clinical variables were selected based on published studies, accessibility, and professional knowledge.^7–11,18,19^ We collected the basic information (gender, body mass index [BMI], smoking, previous history of VTE, etc.), laboratory data (preoperative PSA, postoperative D-Dimer, postoperative PLT, coagulation index, T stage, etc.), imaging results (prostate volume), and treatment measures (operation time, preoperative antiplatelet, neoadjuvant ADT, blood transfusion, lymph node dissection, etc.) from the hospital medical records. Of note, patients with intermediate and high-risk of biochemical recurrence were selectively undergone pelvic lymphadenectomy. If a lymph node dissection was deemed necessary, an extended lymph node dissection was mostly performed. Besides, all patients were scored with Caprini risk assessment on admission to assess the risk of VTE during the perioperative period.^20^ Patients with a caprine score of 3–4 was given mechanical prophylaxis until ambulation, while those with a caprine score more than 5 was given pharmacologic prophylaxis after surgery, according to the recommendations

### Model Building

We first performed univariate logistic regression analysis on candidate variables and then performed multivariate logistic regression analysis on variables with significant differences to select independent risk factors. A nomogram model was accordingly constructed based on the independent risk factors. Meanwhile, to get the optimal model, we also established another evaluation model by package “MASS” and stepwise logistic regression was used to obtain clinical variables for model construction.

After constructing the two new models by using the univariate and multivariate logistic regression analysis (model A) and stepwise logistic regression analysis (model B), we calculated the calibration and discrimination in the primary training dataset.

Receiver operating characteristic curve (ROC) was generated, and the area under the curves (AUC) was used to measure discrimination. Meanwhile, the Brier score, which was calculated as the following formula, was employed to test calibration^21^:

$$ {\text{Brier}}\;{\text{Score}} = \frac{1}{N}\mathop \sum \limits_{t = 1}^{N} \left( {f_{t} + o_{t} } \right)^{2} $$

*N* forecasted instances; *f*_*t*_ forecasted probability; *o*_*t*_ true event.

Then AUC, Net Reclassification Improvement (NRI) and Integrated Discrimination Improvement (IDI) were used as indicators to compare predictive abilities of two new models and Caprini risk assessment (CRA) model. “pROC,” “nricens,” and “PredictABEL” packages were utilized to process the analysis above. The method of Bootstrap was used to self-validate by 1000 repetitions and followed with another internal validation in the testing cohort. The C-index was calculated by use of the RMS package.^22^ Finally, for ease to use clinically, nomograms were constructed for each new model fitted in the above regression methods.

### Statistical Analysis

In this study, variables with more than 30% missing values were discarded, and the missing data were filled by predictive mean matching algorithm. The measurement data were presented in the form of mean with standard deviation or median with interquartile range, and the count data were presented in the form of a number (percentage). The measurement data were compared by two unpaired sample *t*-test, and the count data were compared by chi-square test or Fisher’s exact test. Two-sided *p* < 0.05 was considered as statistical significance. All statistical analyses were conducted through SPSS (version 26.0) and R software (Version: 4.1.0).

## Results

### Baseline and Clinical Characteristics of Patients with Prostate Cancer

Table 1 presents the univariate analysis of baseline characteristics in the whole cohort (n = 351). Among them, 40 (11.4%) patients developed VTE, and the remaining 311 (88.6%) patients who did not develop thrombosis within 30 days after surgery were divided into the non-VTE group.

Compared to the non-VTE group, the VTE group had an older age (69.53 years vs. 64.76 years, *p* < 0.001), a larger prostate volume (68.06 ml vs. 38.59 ml, *p* < 0.001) with a higher PSA level (46.97 ng/ml vs. 37.63 ng/ml, *p* = 0.226), and a higher Gleason score (72.5% with a score ≥8 vs. 30.9% with a score ≥8, *p* < 0.001), which usually indicated a poor prognosis. In addition, the VTE group experienced longer operation time (243.30 min vs. 182.55 min, *p* < 0.001) and more lymph node dissection (70.0% vs. 25.1%, *p* < 0.001). It is interesting to note that, in our study, many patients in the VTE group had a clear previous history of VTE (35.0% vs. 1.3%, *p* < 0.001), and had a higher coagulation index (5.30 vs. 2.32, *p* < 0.001) before surgery (Table 1).

### Risk Factors for Modeling

Two different models were established in our study. To avoid overfitting, factors with *p* < 0.05 in univariate analysis were included in further multivariate regression analysis. In multivariate analysis, Gleason score (*p* = 0.029) and coagulation index (*p* < 0.001) were screened as independent predictors for VTE after RARP (Table 2).

**Table 2:** Variables and coefficients included in stepwise logistic regression model (Model A) and logistic regression with univariate and multivariate model (Model B)

Variable	Model A			Model B		
	Estimate	SE	*p* value	Estimate	SE	*p* value
(Intercept)	28.330	9.180	0.002	− 102.150	39.913	0.010
Age	0.192	0.107	0.073	0.706	0.299	0.018
Prostate volume	0.039	0.023	0.099	0.250	0.107	0.020
Operation time	0.009	0.008	0.285			
Coagulation index	1.696	0.430	0.000	6.856	2.538	0.007
Previous history of VTE	2.144	1.717	0.212	7.182	6.155	0.243
Neoadjuvant ADT				− 11.320	5.032	0.024
Lymph node dissection						
Standard	1.989	2.744	0.469			
Extended	0.389	0.972	0.689			
Blood_transfusion				− 4.582	2.681	0.087
Gleason score ≥8	2.168	0.994	0.029			
Tumor stage				− 9.174	4.112	0.026
Preoperative antiplatelet				− 20.891	2763.889	0.994
PSA				0.057	0.024	0.020
Preoperative APTT				0.450	0.217	0.038
Postoperative PLT				− 0.043	0.022	0.052
Postoperative D-Dimer	0.231	0.191	0.226	0.710	0.316	0.025

*VTE* venous thromboembolism; *APTT* activated partial thromboplastin time; *PLT* platelet; *ADT* androgen-deprivation therapy

Meanwhile, we applied a stepwise logistic regression algorithm using Akaike's Information Criterion (AIC) to select the significant predictors and a combination containing 12 variables is initially enrolled. As a result, age, coagulation index, prostate volume, neoadjuvant ADT, tumor stage, Gleason score, PSA level, preoperative APTT, and postoperative D-Dimer were finally selected for model construction. For all of the above, *p* < 0.05 was considered significant (Table 2).

### Development and Assessment of New Models

**Fig. 1:**
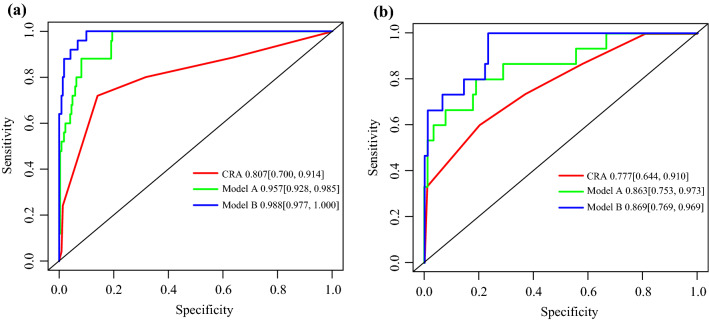
ROC curves of two new models and Caprini scores in the training cohort (**A**) and in the testing cohort (**B**). Model A, univariate and multivariate logistic regression model; Model B, stepwise logistic regression

The ROC was conducted to assess the discrimination performance of established models. Model B was shown to be the best with an AUC of 0.988 (95% confidence intervals [CI]: 0.977–1.000), whereas model A was not inferior, with an AUC of 0.957 (95% CI 0.928–0.985). The AUC of Model A and Model B was both obviously higher than the CRA model (0.807, 95% CI 0.700–0.914). Those results also were confirmed in the testing cohort with AUCs of 0.869 (95% CI 0.769–0.969), 0.863 (95% CI 0.753–0.973), and 0.777 (95% CI 0.644–0.910), respectively (Fig. 1).

**Fig. 2:**
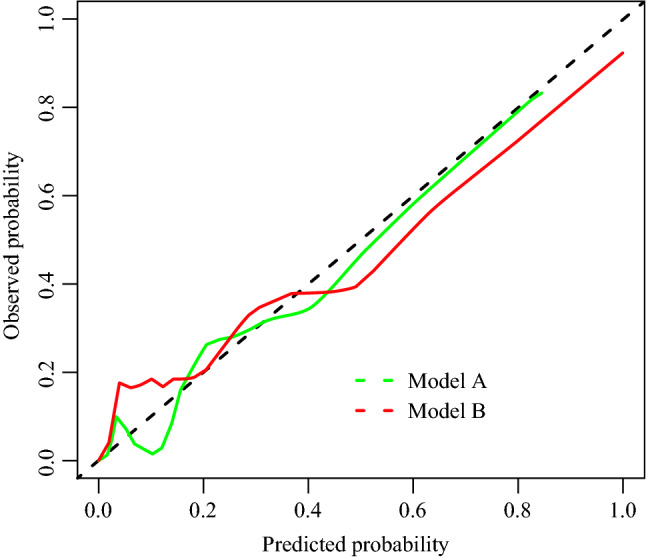
Calibration curve of model A and B. X-axis: risk prediction of VTE in patients. Y-axis: actual diagnosed VTE. The more solid bias corrected line was closed to ideal line, the better prediction capacity. Model A, univariate and multivariate logistic regression model; Model B, stepwise logistic regression

Next, we evaluated the calibration of models A and B in the training cohort (Fig. 2). The Brier score of model A was calculated to be 0.046 (range 0.028–0.063), and the Brier score of model B was 0.025 (range 0.011–0.038), which reflects the good accuracy and consistency of the two new models.

After 1,000 times internal verification with Bootstrap by using R programming language, the C-index of the model A and B were 0.957 and 0.988, which showed that the prediction probabilities of both two new models were consistent with the actual probability, and they had high prediction accuracy.

### Nomogram Visualization

**Fig. 3:**
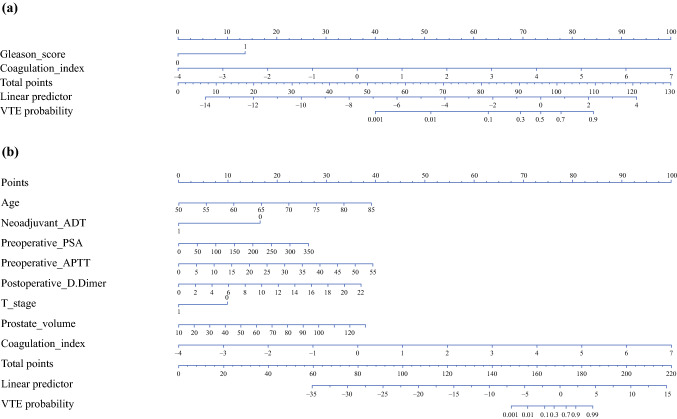
The nomogram obtained from model A (**A**) and model B (**B**). Model A, univariate and multivariate logistic regression model; Model B, stepwise logistic regression

Considering the convenience of clinical applications, the above two models were converted into visual nomograms (Fig. 3). Overall, Nomogram A has an advantage of less variable. Nomogram A (Fig. 3A) contains two variables: Gleason score and coagulation index. Nomogram B (Fig. 3B) contains eight variables: age, prostate volume, coagulation index, neoadjuvant ADT, tumor stage, PSA, preoperative APTT, and postoperative D-Dimer. In general, the higher the total score of the nomogram, the greater the probability of VTE. As a sensitivity analysis with 20% testing data, the further analysis screened out the consistent variables as the presented two nomograms (Supplementary Fig. 1).

### Improvement of Both Models Compared with CRA Model

Furthermore, we accessed the improvement of the prediction accuracy of the two new models compared with the CRA model by using NRI and IDI.^23^ The VTE risk was classified at nodes of 0.2 and 0.4 as cutoff. As results showed, both the prediction and discrimination performance of the two new models were superior to the CRA model (NRI > 0, IDI > 0) with significant statistical differences (*p* < 0.05; Table 3).

**Table 3:** Improvement in prediction abilities of two new models compared with CRA model

	Model A	*p* value	Model B	*p* value
NRI (Categorical) (95% CI)	0.596 [0.393–0.799]	< 0.001	0.700 [0.472–0.928]	< 0.001
NRI (Continuous) (95% CI)	1.406 [1.167–1.645]	< 0.001	1.561 [1.294–1.828]	< 0.001
IDI (95% CI)	0.337 [0.246–0.430]	< 0.001	0.570 [0.445–0.694]	< 0.001

*NRI* net reclassification improvement, *IDI* integrated discrimination improvement

### Decision Curve Analysis for Clinical Application

**Fig. 4:**
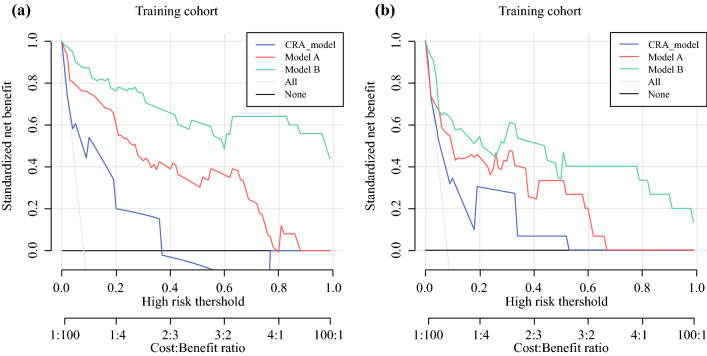
Decision Curve Analysis curve of model A, model B, and CRA model in training cohort (**A**) and testing cohort (**B**). Model A, univariate and multivariate logistic regression model; Model B, stepwise logistic regression

**Fig. 5:**
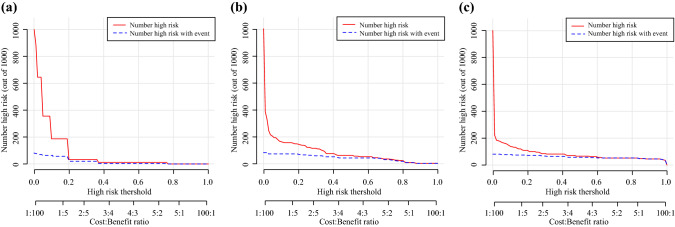
Clinical impact curve of CRA model (**A**), model A (**B**), and model B (**C**). Model A, univariate and multivariate logistic regression model; Model B, stepwise logistic regression

Finally, decision curve analysis (DCA) was conducted to evaluate the clinical usefulness of the two prediction nomograms. The two new models offered a net benefit at the threshold probability between 0.08 and 1 in the training cohort (Fig. 4A). Although the limited sample size of the testing cohort, the two new models were basically the higher line on the decision curve, indicating a higher net benefit of them (Fig. 4B). In addition, the clinical impact curve visualized the estimated numbers who were classified as high risk of VTE and true VTE under the use of the two new models. Compared with the CRA model, the red solid lines of the two new models were closer to their blue dotted lines, suggesting the two new models may predict future VTE events more accurately at each threshold probability (Fig. 5).

## Discussion

The risk of VTE after RARP is not very high,^24,25^ but prostate cancer patients with VTE tend to indicate a poor prognosis and shorter life expectancy.^12^ In addition, they also have an increased risk of recurrent venous thromboembolism and severe bleeding complications.^12^ Therefore, it is important to identify high-risk patients and take early intervention to reduce the incidence of VTE after RARP. Nevertheless, it is not easy to predict the risk of VTE. The tumor was heterogeneous itself, and the risk of VTE was related to the interaction among tumor cells, coagulation system, and clinical characteristics of patients.^12^ A precise predicting tool special for each type of cancer may greatly do a favor to clinicians to predict postoperative VTE. However, at present, most of the references are based on the investigation for risk factors of postradical prostatectomy, still lacking accurate prediction models for individual VTE risks.^24–26^

Our study shows that the risk of post-RARP VTE in patients is mainly related to the following factors: age, coagulation index, prostate volume, neoadjuvant ADT, tumor stage, Gleason score, PSA level, preoperative APTT, postoperative D-Dimer, and Gleason score, which is mostly consistent with previous reports of risk factors for postoperative thrombosis.^9–11^ Among them, Gleason score is a widely used method for histological grading of prostate cancer in current clinical guidelines. Usually, the higher the score, the worse the prognosis.^27^ In addition, prostate volume is associated with the grading and prognosis of prostate cancer.^28^ These results indicate that the increase of VTE is an inherent biological characteristic of some tumors. Research by Cronin-Fenton et al. confirmed that in their study cohort, the VTE risks for the special cancers varied even after adjusting for all the possible confounders.^29^ The risky factor includes hypercoagulable state caused by tumor, vascular damage caused by treatment, surgery, and tumor itself.^30,31^ Noteworthy, cancer patients tend to receive more medical intervention and a forced sedentary lifestyle, both of which increase the risk of venous thromboembolism. Larger prostate volume, neoadjuvant ADT, and advanced tumor stage mainly cause the longer operation time, which also is an important factor affecting the risk of VTE after RARP, in accordance with the findings of Abel et al.^10^ Published researchers have elaborated that excessive operation time would increase stress response, change coagulation status, and increase the risk of thrombosis. In addition, anesthesia time would increase due to prolonged operation time. Thus, the anesthesia would slow down the patient's blood flow, and the anesthetic drug may damage the patient's vascular endothelial cells as well, leading to the occurrence of thrombosis.^32,33^ Tasaka et al. have proved that patients older than aged 60 years have an increased risk of postoperative thrombosis.^34^ Elderly patients usually have higher blood viscosity, thicker intima, impaired cardiopulmonary circulation, and decreased immunity (susceptible to infection), making them more likely to develop thrombosis.^35^ In addition, elderly patients recover more slowly and stay in bed for a longer time, which also greatly increases the risk of thrombosis. It is worth noting that in our study, only 1.3% (4/311) of patients in the non-VTE group had a previous history of thrombosis, compared with 35.0% (14/40) in the VTE group, which showed significant statistical difference in univariate analysis (*p* < 0.001), and suggested that there is a strong correlation between the risk of postoperative VTE and previous history of thrombosis in patients with prostate cancer. Although it may be a strong independent predictor, this factor was not finally included in the model (*p* > 0.05). The reason could be that the two new models constructed by other combined variables would be closer to the actual prediction results. In this study, the median PSA level and ranges are very high when compared with literatures, one reason could be the higher percentage of advanced prostate cancer in China and second could be the neoadjuvant ADT used here. In addition, 11.4% patients developed VTE in this study, which is surprisingly higher than that in previous reports.^5,6^ However, it is plausible considering the greater attention given to postoperative venous thromboembolism prophylaxis in our center and more patients received VTE screening as mentioned above.

Caprini score is a common way to evaluate patients at risk of VTE,^20^ which has been demonstrated in patients undergoing different surgeries, of which urological surgery accounts for 17% of the study population.^36^ There are more than 30 indicators used to assess the risk of thrombosis of patients; however, it seems too complicated to use, not conducive to clinical fast decisions. In addition, due to the uniqueness of prostate cancer, Caprini score’s universal standards may not be very appropriate for the risk assessment of prostate cancer. Furthermore, the models obtained by stepwise regression and U&M regression in this study have respectively eight and three variables, which greatly simplifies the risk assessment process of VTE. Model A has an advantage of less variable. The AUC of the two new models is significantly higher than the CRA model, besides, both NRI and IDI > 0, which shows better predictive capabilities. The advantages of the visualization of the nomogram also improve the convenience of clinical applications and do a favor to clinical evaluation and management, early thromboprophylaxis would be suggested when a risk of VTE was calculated by the nomogram. Notably, the applied statistical methodology could be overfitted due to the limited study size.

Indeed, our study still has some limitations of the following aspects. First, the prediction models in one clinic does not necessarily have to be useful in another. One way forward is to look for causal associations and to let each center make its own prediction models with the support of evidence for causality. Second, this is a retrospective, observational, single-center, retrospective study with limited study size, which is easy to produce sampling error and needs to be verified by the data of larger samples. Lastly, by use of a nomogram, which kind of thromboprophylaxis should be taken to maximize the benefits still needs further study.

## Conclusions

By the retrospective analysis of prostate cancer data from our center, two sets of prediction models were developed. Both two new models have good prediction accuracy and are superior to the well-known CRA model in our clinic. Model A has an advantage of less variable. This easy-to-use nomogram may help to rapid clinical decision-making for urologists and early thromboprophylaxis in high-risk groups that ultimately benefit patients, which encourage the researcher to make a more stable and universal prediction nomogram of VTE for RARP.

## Supplementary Information

Below is the link to the electronic supplementary material.

Supplementary file1 (TIF 160 kb)

Supplementary file2 (TIF 2262 kb)

Supplementary file3 (DOCX 33 kb)

## Data Transparency

All data and materials as well as software application or custom code support the published claims and comply with field standards.
